# Density-Based Descriptors of Redox Reactions Involving Transition Metal Compounds as a Reality-Anchored Framework: A Perspective

**DOI:** 10.3390/molecules26185541

**Published:** 2021-09-13

**Authors:** Daniel Koch, Mohamed Chaker, Manabu Ihara, Sergei Manzhos

**Affiliations:** 1 Centre Énergie Matériaux Télécommunications, Institut National de la Recherche Scientifique, 1650 Boulevard Lionel-Boulet, Varennes, QC J3X 1S2, Canada; mohamed.chaker@inrs.ca; 2 School of Materials and Chemical Technology, Tokyo Institute of Technology, Ookayama 2-12-1, Meguro-ku, Tokyo 152-8552, Japan; mihara@chemeng.titech.ac.jp

**Keywords:** oxidation, transition metal compounds, density functional theory, charge self-regulation, oxygen redox

## Abstract

Description of redox reactions is critically important for understanding and rational design of materials for electrochemical technologies, including metal-ion batteries, catalytic surfaces, or redox-flow cells. Most of these technologies utilize redox-active transition metal compounds due to their rich chemistry and their beneficial physical and chemical properties for these types of applications. A century since its introduction, the concept of formal oxidation states (FOS) is still widely used for rationalization of the mechanisms of redox reactions, but there exists a well-documented discrepancy between FOS and the electron density-derived charge states of transition metal ions in their bulk and molecular compounds. We summarize our findings and those of others which suggest that density-driven descriptors are, in certain cases, better suited to characterize the mechanism of redox reactions, especially when anion redox is involved, which is the blind spot of the FOS ansatz.

## 1. Introduction

Chemical reactions, including formation reactions of compounds—whether molecular or solid—are essentially phenomena of bond reformation. They always involve redistribution of charge. As a good approximation, in most cases, it is sufficient to consider redistribution of the valence charge originating from the valence shells of constituent atoms [1,2,3]. In this process, some atoms may lose and some may gain charge in their vicinity, being so-called oxidized and reduced, respectively. In textbooks, one can see oxidation and reduction defined as loss and gain, respectively, of *one or more electrons*. Some define them as change in oxidation *number*, and some as *actual or formal* transfer of electrons [4,5]. The formal transfer of an integer number of electrons referred to here is based on the concept of formal oxidation state (FOS) widely used in theory and in applications [6,7]. We will present a formal recapitulation of the FOS formalism in Section 2.1.

This language implies charge quantification. However, actual transferred charges are only quantized in long-range charge transfer. Charge transfer that is relevant for the formation of compounds and chemical reactions (not involving dissociation into ionic species) is not long-range and is *not* subject to the quantization postulate [8,9,10,11]. It immediately follows that the ansatz of integer charge counting is formally handicapped from the onset and that its range of applicability, and the insights that it can generate, should be carefully assessed against the actual (change of) electron density topology and other observables. The concept of FOS predates modern quantum and computational chemistry, dating back to the 1930s [12]. It introduces a set of rules to assign electrons to atoms in a compound based on simple molecular orbital considerations and the ionic approximation (which we recapitulate below) [7]. It allows one to walk away from the complexity of continuous electron density and create an easy-to-use scheme with which to characterize matter. Formal oxidation states are fictitious by definition.

The FOS picture has fruitfully served, in particular, due to its ability to predict the compositions of inorganic compounds with high accuracy. This ultimately has to do with FOS being defined from the interactions of the valence states of atoms (see below), where the resulting valence states of compounds may be qualitatively correctly inferred, even if the actual redistribution of charge in the compound is not correctly reflected. To give an illustrative example, consider Ti ions. Laser irradiation of titanium can create plasma in which Ti ions with different charge states can be reliably detected, including 0, +1, …+4 |*e*|, as well as higher charge states [13,14]. These actual charge states of isolated Ti ions, including Ti^4+^, are equal to their formal oxidation states. In the Ti^4+^ isolated ion, the Ti valence shell is fully ionized. The formal oxidation state of Ti in the titanium dioxide is also +4, even though there is a significant valence charge remainder on the Ti core [15]. At least in this case, both the actual and the FOS indicate Ti oxidation, but differences in FOS and actual charges can be substantial enough to paint qualitatively different pictures and they are not limited to transition metal (TM) compounds which are the focus of this perspective. An example is Ca(CO)_8_, in which the Ca atom, which *is* oxidized, has a FOS of zero [16,17]. Other examples are given in Refs. [15,18,19,20,21,22,23,24,25,26,27,28] and below.

In 1948, Linus Pauling formulated “*the postulate of the essential electrical neutrality of atoms: namely, that the electronic structure of substances is such as to cause each atom to have essentially zero resultant electrical charge, the amount of leeway being not greater than about* ±1/2*, and these resultant charges are possessed mainly by the most electropositive and electronegative atoms, and are distributed in such a way as to correspond to electrostatic stability. According to this postulate the description of the barium oxide crystal as consisting of an arrangement of ions Ba^++^ and O^−−^ would be a poorer approximation to reality than its description as a regular arrangement of atoms Ba and O, with each atom forming two covalent bonds … These bonds have enough ionic character to give a small positive charge to each barium atom and a small negative charge to each oxygen atom*” [29]. Modern computational chemistry essentially has confirmed this proposition for transition metal compounds, although it was not until 80 years after Pauling’s prediction that charge self-regulation in TM compounds was firmly established [26].

Correctly reflecting the actual charge redistribution in a compound or during a reaction is, however, critically important for the understanding of the mechanism of bonding in compounds and the mechanism of redox reactions, i.e., reactions with significant charge transfer. This is all the more important as redox reactions are at the core of the mechanism of operation of multiple technologies, most notably, many of the technologies developed for sustainable energy conversion and storage and environmental cleanup. Materials used in technologies from photocatalytic synthesis [30,31,32] and water splitting [33,34,35] to environmental cleanup [36,37] to solar cells [38,39,40] to smart windows [41,42] to electrochemical batteries [43,44,45] are often based on transition metal oxides (TMOs), in which the oxidized TM centers are reduced by photoelectrons or electrons donated by dopants. The most performant cathode materials for metal ion batteries—including Li ion batteries as well as post-Li technologies such as Na, Ca, Mg, or Al ion batteries—are TMOs [46,47,48,49], in which the insertion mechanism of lithiation, sodiation, etc., is based on charge donation by the inserted working metal (Li, Na etc.) atom and reduction of the TM center. (Although, in an actual battery, it is the cation of the working metal that is inserted into the oxide from the electrolyte, while the electron comes from the external circuit, the end result of the insertion is a neutral system in which valence electrons of the working metal reduce TM centers.) 

Metal ion batteries present a practically important example of why descriptors of charge distribution that give justice to the actual degree of oxidation and reduction are needed for correct mechanistic understanding of the charge–discharge process and, ultimately, for rational design of new and high-performance active electrode materials. Oxygen redox in battery materials has been acknowledged relatively recently and presents a promising direction for increasing specific capacity [50,51,52,53]. While oxygen redox is most prominent in Li-excess materials, a degree of oxygen redox in terms of real-space charge density transfer can be observed in all TM oxides (the terms electron density and charge density will be used interchangeably throughout this work) [54,55]. It is clearly seen in DFT calculations from the analysis of valence electron density and cannot be written off as an artefact of DFT calculations, as DFT densities are in good agreement with available soft X-ray and electron diffraction experiments [56,57]. In the case of, e.g., Ti-based compounds, the “Ti^4+^” language of FOS implies no further oxidation of Ti and no further reduction of O, and misses oxygen redox outright, while density-based descriptors have no trouble capturing these.

In this perspective, we therefore focus on the electron density-based description of redox reactions involving transition metal compounds as a reality-anchored framework which permits mechanistic understanding of such reactions without the distortions induced by the FOS picture. We summarize our findings and those of others which suggest that density-driven descriptors are more apt to give justice to the mechanisms, especially when anion redox is involved, which is the blind spot of the FOS ansatz. Contrary to one-electron wave functions, the density is an observable. However, and separate from the reliance on FOS, non-observable electronic states, in particular single-electron Kohn-Sham states, are still important for the mechanistic understanding of redox reactions and of mechanisms in TM compounds in general; indeed, an important computational tool is the partial or projected density of states (PDOS) routinely used to parse effective contributions of atomic states of different angular momenta to the redox reaction. We also show that density-based analysis allows uncovering shortcomings in the common (spherical approximation-based) approach to PDOS and a more significant contribution of anionic redox than is commonly perceived.

## 2. Oxidation and Charge Self-Regulation

### 2.1. Formal Oxidation State Ansatz

**Figure 1:**
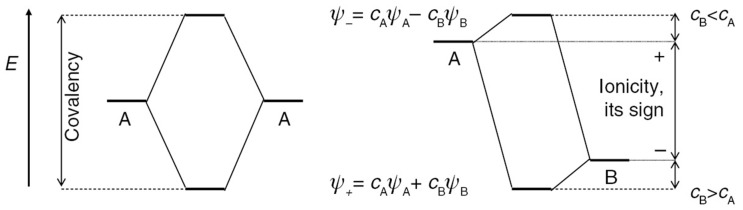
Simple MO schemes for two species A and B with electronic states *ψ_A_* and *ψ_B_*. Left: formation of a homonuclear bond without polarization. Right: formation of a polarized heteronuclear bond with two resulting states *ψ_−_* and *ψ_+_* as a result of the linear combination of atomic orbitals with coefficients *c*_A,B_. Reproduced with permission from Ref. [7], used in accordance with the Creative Commons Attribution (CC BY-NC-ND 4.0) license (https://creativecommons.org/licenses/by-nc-nd/4.0/, accessed on 20 August 2021).

As previously mentioned, the FOS ansatz is based on the ionic approximation, assigning each electron of a compound to one of its constituting atoms. In the recommended IUPAC definition [6,7], the idealized fully ionic distribution of electrons between atomic centers is achieved by counting all one-electron states that can be attributed to a certain species and subtracting this number from the number of electrons of its neutral atom. While for homonuclear bonds the shared electrons are, by definition, equally divided between the two species, for heteronuclear bonds the electronic states are assigned to the species with the largest contribution to the respective state. In a simple molecular orbital (MO) picture like the one shown in Figure 1, this would correspond to the species whose contributing atomic orbital is closest in energy to the respective MO or, alternatively, has the largest MO coefficient *c* in a linear combination of atomic orbitals (LCAO) picture. 

For highly ionic compounds with one of the MO coefficients for each one-electron state being significantly larger than the other(s), the bound ions show a high degree of similarity to their free-ion counterparts in terms of energy eigenvalues of orbitals, but also in the local distribution of charge density around the nuclei, which can be, for example, measured by X-ray diffraction experiments [8,56,57]. For covalent compounds, on the other hand, the one-electron states are delocalized to a certain degree, with non-negligible contributions from more than one species and a larger deviation from one-electron energies of the free ions and their charge distribution around the nuclei. Although, for redox processes involving covalent compounds, FOS are occasionally used for the rationalization of redox processes, e.g., in organic battery materials during cycling [58], the hybridization between different atomic orbitals is widely acknowledged in literature and the discussion of oxidation and reduction processes is often centered around whole functional groups rather than specific atoms [59,60,61]. For redox reactions involving ionic compounds, on the other hand, the FOS concept has proven itself as a powerful tool, condensing the complexity of electronic (and ionic) structure changes during chemical reactions involving electron transfers into effective properties assigned to individual ionic species. However, full ionicity constitutes an idealized limit and real (heteronuclear) compounds exist on a spectrum of varying degrees of ionicity or covalency. This is especially important to keep in mind for transition metal compounds, which are often regarded as ionic but have been shown to possess a significant covalent character [62,63]. This has been long acknowledged, as Linus Pauling wrote already in 1948: *“I doubt whether the ferrous ion and ferric ion, and similar ions of the transition elements, exist in chemical substances. I think instead that the atoms of iron in all ferrous and ferric compounds form covalent bonds in such a way as to remain essentially neutral”* [29].

Oxidation states are often related to experimental observables, which allow embedding redox reactions into the FOS framework. An often-cited experimental reference for oxidation states is X-ray photoelectron spectroscopy (XPS), where core electron energies’ states are related to the FOS of the ion. It should be noted, however, that XPS reference energies are obtained from compounds with a matching FOS of the corresponding species, e.g., for the assignment of 2p_3/2_ peak of the above mentioned “Ti^4+^” cation in titanium(IV) compounds, its energy (which is also dependent on the chemical environment of titanium) is compared to the one measured in TiO_2_, not an actual isolated Ti^4+^ cation [64,65,66]. While XPS spectra allow the assignment of a postulated FOS based on the postulated FOS of a reference compound, oxidation states can neither be measured directly, nor do XPS peaks provide any information on the actual charge distribution around the corresponding species or its atomic contributions to hypothetical one-electron states. The latter can furthermore not be probed experimentally anyway, since the measurement of electronic transitions always occurs between two states of the full many-body wave function, despite often being rationalized as the transition between orbitals. Similar arguments hold for the assignment of FOS via, e.g., X-ray absorption near-edge structure, Mößbauer spectroscopy, or electron paramagnetic resonance. In practice, formal oxidation states are a convolution of material properties which can—but do not need to—resemble the properties of free ions.

While FOS can be determined ad hoc based solely on a set of empirical rules [7], quantum chemical calculations are frequently invoked to gain additional insight into electron transfer reactions and the charge distribution within compounds [67,68,69]. Population analysis schemes allow the allocation of charges to individual atoms based on the computed wave function or electron density of (post-) Hartree–Fock and DFT calculations beyond integer charge assignment, in order to provide a more nuanced picture of the partial charges of different ionic species. These analysis methods are generally divided into three categories [70]: partitioning schemes based on the projection onto basis functions, partitioning of the charge density into atomic domains, or population analysis by fitting the electrostatic potential (ESP). The ESP methods, e.g., the restricted ESP model [71] and the distributed multipole analysis [72], will not be discussed in the following due to their low relevance for the subject of this perspective. It has been pointed out before that the FOS concept, in contrast to the partitioning schemes based on ab initio results, does not aim to reflect the valence charge density distribution between ionic centers accurately, but rather provides information on how many electrons are actively involved in bonding [73]. Nevertheless, it is necessary to be aware of the intrinsic limitations of the FOS concept in order to understand to what extent mechanistic rationalizations can be based on it and where more intricate approaches to charge analysis become necessary.

### 2.2. Relation to Projection Approaches

Projection approaches are based on the distribution of the electrons occupying each orbital of the compound between the atomic centers contributing basis functions to the respective orbital. The commonly used Mulliken population analysis [74,75,76,77], for example, is based on pairwise products of LCAO coefficients multiplied by the overlap of the respective atomic orbitals, dividing shared electrons (off-diagonal elements of the population matrix) equally between the two involved atoms in order to obtain the electron population *p* on an atom A [70]:

(1) $$p_{A}^{\mathrm{proj}}=\Sigma_{\alpha \in A}^{M_{\mathrm{basis}}}\Sigma_{\beta }^{M_{\mathrm{basis}}}\underset{D_{\alpha \beta }}{\underset{︸}{\left(\Sigma_{i}^{N_{\mathrm{orb}}}n_{i}c_{\alpha i}c_{\beta i}\right)}}S_{\alpha \beta }$$

with basis set size *M*, number of orbitals *N*, occupation numbers *n*, and overlap matrix elements *S*; the population matrix elements are *D_αβ_S_αβ_*. However, this equal distribution is chosen arbitrarily and is, in many cases, counter-intuitive, as electrons in polar bonds would be rather localized on the more electronegative atom. In addition, the Mulliken population analysis can yield physically unsound results, such as negative off-diagonal elements of the population matrix, occupation numbers larger than two, or no conservation of electric multipole moments. While the additional computational cost of obtaining Mulliken, or the closely related Löwdin charges [78], is negligible, these partitioning schemes are also known to suffer from a severe basis set dependence with the obtained charges not converging with increasing basis set completeness. Despite these drawbacks, the Mulliken population analysis in particular is still frequently employed to rationalize charge transfer processes [79,80,81]. While originally developed from molecular wave functions, the concept can be easily transferred and applied in extended systems as well.

In periodic systems, the molecular orbitals of compounds, as they were discussed above, transform into continuous energy bands, which can be occupied or unoccupied. The electronic states are *k*-point (Brillouin zone) dependent and their relative energies ϵ are represented in the compound’s density of states (DOS) *D*(*E*), dependent on the energy *E*, which replaces the molecular orbital scheme for solids:

(2) $$D\left(E\right)=\sum_{i}\delta \left(E-\epsilon_{i}\right)$$

In order to reflect the dominant atomic contributions to the DOS, which is commonly used to support the assignment of reduced or oxidized species in solid-state redox reactions, in analogy to the largest MO contributions in the zero-dimensional case, the DOS can be projected onto atomic basis functions $\chi_{A}$ to yield the site- (or by extension, species-) projected DOS *D*_A_(*E*):

(3) $$D_{A}\left(E\right)=\sum_{i}\sum_{A}{\left|\langle \chi_{A}|\psi_{i}\rangle \right|}^{2}\delta \left(E-\epsilon_{i}\right)$$

with the *k*-dependent crystal orbitals ψ. For computations with localized basis functions, the expression in Equation (3) bears similarity to the definition of the diagonal elements of the Mulliken population matrix; for plane-wave bases, χ is in general a suitably chosen localized projection function in a confined spatial domain. In the context of transition metal compound chemistry, these localized projections generally lead to certain ambiguities of the resulting PDOS representation, as will be further elucidated in Section 3.4.

Other than projection methods, density-based population analysis approaches are significantly less sensitive to the basis set choice and are based on the experimentally observable charge density ρ. These methods partition charges in real space in contrast to the partitioning of the Hilbert space spanned by basis functions [70]. Atomic populations are the result of the charge density integration in certain atomic domains Ω_A_ of the general form:

(4) $$\;p_{A}^{\mathrm{dens}}=\int_{\Omega_{A}}^{\;}w_{A}\left(\vec{r}\right)\rho \left(\vec{r}\right)d\vec{r}\;$$

with $w\left(\vec{r}\right)$ being weighting functions for the atomic domains. While previously, ambiguity in the population definition was introduced by the partitioning of the projected charges, it is now the partitioning of space which is method-dependent. Several partitioning algorithms exist. Voronoi charges [82,83] are based on the tessellation of real space into domains closest to each nucleus, which define the extent of each Ω_A_ in which the deformation densities are integrated with $w\left(\vec{r}\right)=1$. For Hirshfeld charges [84], $w\left(\vec{r}\right)$ are defined by the ratio of the atomic density of A and the superposition of all atomic densities, while Ω_A_ spans the whole space. The most prominent density-based method, however, is the quantum theory of atoms in molecules (QTAIM) approach proposed by Bader [85,86,87,88], with $w\left(\vec{r}\right)=1$ and the atomic domains confined by so-called zero-flux (ZF) surfaces. These surfaces ${\vec{r}}_{\mathrm{ZF}}$ are the boundaries of the atomic domains for which the gradient perpendicular to the surface vanishes

(5) $$\nabla \rho \left({\vec{r}}_{\mathrm{ZF}}\right)\cdot \vec{n}\left({\vec{r}}_{\mathrm{ZF}}\right)=0$$

with $\vec{n}$ being the surface normal. The ZF surfaces coincide with the density minima along the interatomic bonding axes and hence correspond to the most intuitive way in which charge density basins would be defined from its topology. It has been shown before that the topological QTAIM partitioning captures the density contributions, which can be clearly assigned to the transition metal species in transition metal oxides, while Voronoi and Hirshfeld populations often tend to be significantly larger [15,70]. Although the partitioning of space is in principle arbitrary, Bader charges provide a reliable answer to the question of *where* electrons accumulate during redox reactions, taking into account the heterogeneous shapes and extent of the charge density distribution around ions. 

### 2.3. Charge Self-Regulation

**Figure 2:**
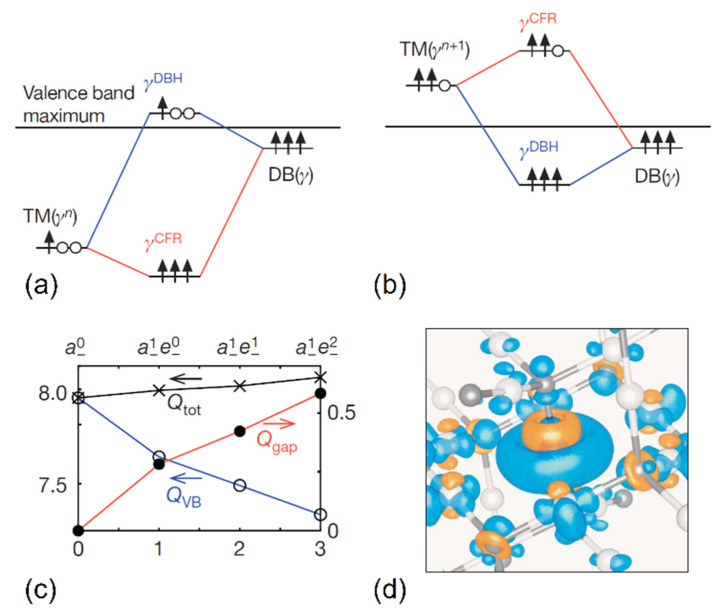
Energy level scheme examples for a transition metal compound with (**a**) charge *q* and (**b**) charge *q* − 1 around the valence band maximum of the bulk. Upward arrows indicate occupied states of the TM, the ligand dangling bonds (DB), the TM-assigned crystal field resonance (CFR) hybrid orbitals, and the ligand-assigned hybrid (DBH) levels; empty circles indicate the corresponding unoccupied states. TM and DB states are chosen from the same irreducible representation *γ*. The integrated charges *Q* around a Co dopant in Cu_2_O are plotted against the total charge of the simulation cell in (**c**) and decomposed into low-lying valence band (VB) and high-energy gap state contributions (in insulators). The corresponding charge density difference around Co between the neutral and the +1 charged system is shown in (**d**), with positive density differences represented by blue, negative ones by orange isosurfaces (isovalue −0.003 |e| Å^−3^). Adapted with permission from Springer Nature Customer Service Centre GmbH: Springer Nature [26], Copyright 2008.

Although oxidation and charge states of ions in their compounds are themselves used, e.g., to explain TM complex geometries or in the construction of force fields, respectively, it is their *change* during chemical reactions which is of interest in most cases. In the fully ionic limit, the anionic and cationic wave functions do not overlap, and any addition or removal of charges to or from the system only affects the electronic structure close to one of the ionic species; FOS changes correspond then to the changes in real-space ionic charges. In the case of (partially) covalent compounds in which hybridization between the atomic states occurs, however, the charge state variation of the ions depends on the ionic contributions to the newly (de-)occupied states, but also on any coefficient shifts between cations and anions in the states formally not partaking in the redox process itself. This means that in (partially) covalent compounds, such as those formed by TMs, any occupation of TM-assigned states (resulting in a decrease of its oxidation state) can, in principle, be alleviated in real space by the loss of TM contributions to lower-lying states, and vice versa. Such a behavior was reported by Raebiger et al. from DFT calculations for transition metal compounds [26], where charge doping was found to increase the energy of TM states relative to the ligand orbitals, resulting in a depletion of TM contributions to low-energy states while they increase for the frontier orbitals. This is illustrated in the MO schemes in Figure 2a,b with example TM and ligand orbitals of the same irreducible representation, where the ionic TM and TM-assigned MOs shift upwards in energy upon reduction. This creates energetically high-lying TM states in the DOS of the bulk compound, while TM contributions shrink for the lower-lying MOs and the valence band becomes more ligand-dominated. 

Consequently, the integrated charge density around TM centers remains nearly constant over a wide range of oxidation states, as was corroborated in Ref. [26] and several other computational studies since [22,23,24,27]. In Figure 2c, the charge stability is shown on the example of a Co dopant in Cu_2_O, where the charge density increase due to TM gap states, and the charge loss from valence band states becoming less TM-like, offset each other, keeping the integrated local charge nearly constant, while the dopant’s FOS is changing with increasing total charge of the system. In real space, this results in charge density losses and gains around the TM center, as shown in the density difference plot in Figure 2d. A significant charge decrease can be observed around the TM center in regions previously hybridized with ligand orbitals, while for other TM d states (with different angular momentum quantum numbers) and around the ligands themselves a charge density gain is found. This effect was described as *charge self-regulation* of transition metal ions by Raebiger et al.

**Figure 3:**
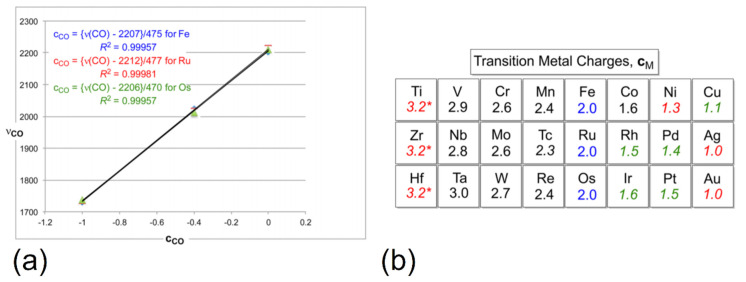
(**a**) Reported average CO stretch frequencies ν for different Fe (blue), Ru (red), and Os (green) carbonyl complexes plotted against their average CO ligand charge c, computed based on a TM charge of +2. (**b**) Average CDVR charges of d block elements relative to the postulated Fe^2+^ reference. Charges obtained from limited datasets are given in green italics, those from a single data point in red italics, and the values for the group 4 elements Ti, Zr, and Hf are derived from the respective row trends. Adapted with permission from Ref. [27]. Copyright 2017 American Chemical Society.

While the relative stability of TM charges regardless of FOS is predominantly discussed in the framework of ab initio calculations, it can be deduced from experimental data as well. Molecular TM complex ligands like CO exhibit a characteristic infrared (IR) signature, which is sensitive to the chemical environment of the molecule. Reduction of CO results in the occupation of antibonding π* MOs and hence a lowering of the C-O bond strength and stretch frequency, causing a red shift of the characteristic CO stretch IR signal. While main group metal carbonyl complexes have been reported [89,90,91], metal–CO bonds are greatly stabilized by metal d orbitals which participate in the formation of bonds with covalent character [17,92]. The TM d orbitals are crucial for the TM carbonyl complex stability and the enhanced covalency of the bonds due to d contributions mitigates the red-shift of the CO stretch frequency, which correlates with the amount of charge transferred between metal and CO ligands (which is the higher the more ionic the bonding) [16]. Therefore, the relative ionicity and charge transfer between TM central ion and ligands in different carbonyl complexes can, in principle, be determined by a comparison of their measured average CO stretch frequencies. Wolczanski analyzed the reported values for TM carbonyls in literature to compute the relative charge states of TM cations in these complexes using the charge distribution via reporters (CDVR) approach [27]. Since no reference for the actual ionic charges in TM carbonyls is available, a Fe charge state of +2 was postulated in iron carbonyls to enable a relative comparison across all compounds. Using the fixed Fe charge, average CO ligand charges were computed and related to changes in the CO stretch frequency. As shown in Figure 3a, this approach leads to a linear relationship between the average CO charges and reported stretch frequencies, allowing for the use of a simple empirical expression to compute transition metal charges in all TM carbonyls. An approximately constant charge state of Fe in the considered iron carbonyl complexes was later confirmed from DFT-derived Bader charges, and a linear relationship between ab initio CO ligand charges and their literature stretch frequencies was found theoretically as well, but with a charge offset of approximately 1 |*e*| due to the overestimation of the CDVR Fe charge reference relative to the DFT results [22]. In accordance with the theoretically found charge self-regulation of TM ions, the CDVR charges do not vary strongly with the FOS of the metal, and hence average TM charges could be computed for all carbonyls, shown in Figure 3b. As can be seen, the charges within a group vary only slightly, while the ionicity of the TM-CO bonds decreases with ascending group number as one would expect. It can also be seen that the average TM charges occur only in a range between ~1 |*e*| and ~3 |*e*|, much narrower than the range of possible oxidation states in TM compounds and similar to the commonly found range of DFT-derived Bader charges in different TM compounds (some of which will be presented in the following sections).

Overall, theory predicts nearly constant local charges around TM cations in their compounds irrespective of FOS, which is reflected in the experimentally observed vibrational frequency changes of charge reporter ligands bound to transition metals. The latter also demonstrates how an elegant rationalization of certain observable TM compound properties can be obtained when charge distribution and transfer is discussed in the framework of partial ionic charges rather than FOS. The following sections will provide a representative summary of theoretical studies utilizing TM ionic charges derived from electron density, showcasing how such a shift of perspective can benefit the understanding of redox processes and uncover aspects which remain otherwise masked by the approximations made in the FOS picture. 

## 3. Density-Based Descriptors as Observables-Based Framework

### 3.1. Charge Distribution in Titanium Dioxide and Its Changes upon Reduction

Already in the year 2000, and before the work of Raebiger et al. on charge self-regulation, Christensen and Carter, in a DFT study, reported significant covalent character of bonds in ZrO_2_ and concluded that in ZrO_2_ *“Zr is likely to be Zr(II) like”* in spite of the formal oxidation state of Zr(IV) [19]. This was rationalized from the perspective that it is easier to remove Zr(5s) electrons from the Zr atoms, while the ionization potential for the Zr(4d) electrons is higher. It is well known that various atomic charge assignment schemes, including density-based schemes and in particular Bader charges, deviate significantly from the formal charges. For basis-dependent approaches, this is not surprising, but for approaches based on the topology of the electron density, this deviation, while natural and method-dependent, deserves to be analyzed for better insight into the mechanism of redox processes. 

For an in-depth analysis we turn to TiO_2_. Titanium dioxide is a widely studied TMO in theoretical and applied literature. It is used as a functional material (material providing the key or defining functionality of the device) in, among other, solar cells (dye-sensitized and perovskite) [93,94,95], in metal-ion batteries [96,97,98], or in photocatalytic systems [99,100,101]. In all these applications, redox reactions involving the change in charge state of Ti as well as O are at the core of the functionality. In the literature, both applied and theoretical, the rationalization of these reactions, and therefore the mechanistic understanding of the relevant technologies, are typically based on FOS. It is understood to be based on the reduction of Ti^4+^ to Ti^3+^ by either the electrons donated by the dopant, photoexcited electrons, or injected electrons, depending on the technology of interest. The rigidity of the FOS picture leaves, in particular, no room for conceptual description of oxygen redox.

**Figure 4:**
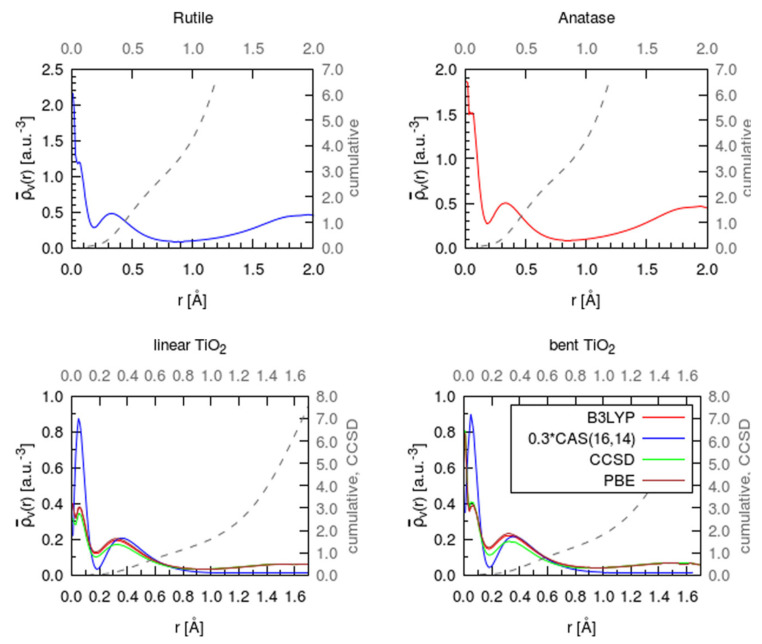
Top panels: spherically averaged valence electron density around the Ti atom of a periodic rutile and anatase structure calculated with PBE. The spatial coordinate *r* corresponds to the radial distance from one Ti center. The O atoms are located at *r* = 1.96 A in rutile and *r* = 1.95 and 2.01 Å in anatase. Bottom panels: spherically averaged valence electron density of a linear and bent TiO_2_ molecule obtained with B3LYP, CAS(14,16), CCSD, and DFT PBE. The CAS(16,14) curve is scaled by a factor of 0.3 for a better comparison. The gray, dashed lines indicate the cumulative numbers of electrons within the spheres of corresponding radii. Adapted with permission from Ref. [15]. Copyright 2017 American Chemical Society.

In Ref. [15], the distribution of the valence electron density in TiO_2_ around the Ti center was studied directly. In Figure 4, we reproduce the spherically averaged valence electron densities around Ti in rutile and anatase TiO_2_ as well as in TiO_2_ molecules in two geometries: linear (mimicking the arrangement in solid titanium dioxide) and equilibrium bent configurations. The molecules were studied to provide a comparison between wave function methods and GGA and hybrid functional flavors of DFT, and to confirm that GGA DFT (used in solid state simulations) provides trustable results. We also note that there is decent agreement with charge densities around Ti in a TiO_6_ environment measured by X-ray/electron diffraction and computed by DFT [56,57]. Also shown in Figure 4 are cumulative charges within the spheres of a corresponding radius around Ti. The cumulative charge reaches one electron within a radius of approximately 20% of the Ti−O distance in anatase and rutile (which is close to 2 Å). This one electron charge cannot be assigned to any other atom but the Ti. It is located well within the QTAIM basin of Ti (delimited by the zero-flux surface). The Bader charge within the basin is about +2.5 |*e*|. This testifies to a significant degree to the covalency of bonding in titanium dioxide. We also note that Phillips fractional ionicity of TiO_2_ (based on Pauling electronegativity) is 0.6 which implies a significant covalent character, compared, e.g., to 0.9 for LiF whereby Li is practically fully ionized [102]. The covalency of the bonding implies a possibility of oxygen reduction which is conceptually excluded in the FOS picture.

**Figure 5:**
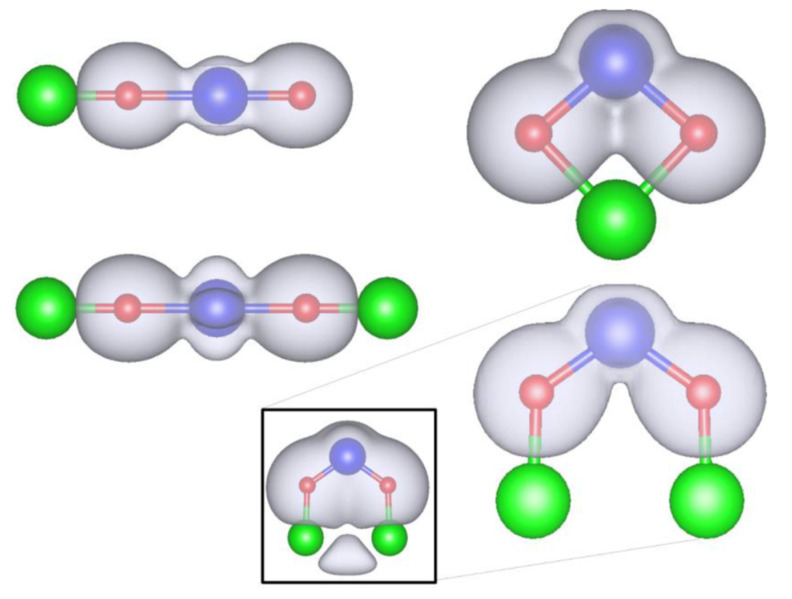
Geometries of four optimized lithiated TiO_2_ molecules. From top left to bottom right: LiTiO_2_ (linear), LiTiO_2_ (bent), Li_2_TiO_2_ (linear), Li_2_TiO_2_ (bent). Isosurface plots of the charge densities in every molecule (isosurface value: −0.03 a.u.) are shown in grey. Ti atoms are represented as blue, oxygen as red, and lithium as green colored spheres. The inset shows an isosurface plot with a lower isovalue (−0.006 a.u.) for bent Li_2_TiO_2_ in order to visualize the non-nuclear attractor (bond) between the two Li ions. Reproduced from Ref. [22], used in accordance with the Creative Commons Attribution (CC BY 3.0) license (https://creativecommons.org/licenses/by/3.0/, accessed on 20 August 2021).

The full ionization of alkali atoms such as Li and Na is also correctly reflected in their Bader charges. This includes ionization upon insertion into TMOs (and other types of materials), including TiO_2_, in which case Bader charges on the order of 0.8–1.0 are routinely reported [22,103,104,105,106,107]. That is, the Bader charge, a density-based measure, can correctly identify the degree of ionicity of bonding or the degree of ionization of an atom. This is in contrast to Mulliken and tessellation-based schemes. For example, a Mulliken charge of Li in Si, which is fully ionized, can even be negative (although the Mulliken population analysis correctly shows loss of s electrons) [108]. Li attachment to a TiO_2_ molecule is also an instructive case which was studied in Ref. [22]: in the LiTiO_2_ complex, the Li is fully ionized (Bader charge of 0.98), and its 3s electron is donated partially to Ti (about half an electron) and partially to the two oxygen atoms (each gaining about a quarter of an electron). The second attached lithium, forming Li_2_TiO_2_, remains fully ionized in a linear LiOTiOLi configuration in which it donates about 0.4 of its valence electron to Ti and the rest to O atoms whose Bader charge passes from −1.22 in linear OTiO to about −1.75 in LiOTiOLi. There is thus significant contribution from oxygen which is possible precisely because both Ti and O have charge states that are significantly lower than their formal oxidation states. Besides the linear configuration, Li attachment to a bent TiO_2_ molecule was also studied. The linear and bent Li_1,2_TiO_2_ molecules are shown in Figure 5. In the case of the bent configuration, the attachment of a second Li creates a bond between the two Li atoms (which can be confirmed by electron density accumulation between them). The additional electron donation to TiO_2_ upon attachment of the second Li atom is only about 0.1 |*e|* and the Li pair keeps about one electron charge (average charge per Li of +0.54). 

It is instructive to compare the densities around Ti in Ti compounds to the densities of free Ti^n+^ ions, which was also performed in Ref. [15]. In Figure 4, one observes the remaining contributions not only from 3d electrons (peak around *r* = 0.4 Å) but also from 4s electrons (peak close to the nucleus). In contrast, in free Ti^3+^ only d contributions are seen, while in Ti^2+^ (as, of course, also in Ti^+^ and Ti^0^) one observes both 3d and 4s contributions [15]. The Bader charge on Ti of less than +2.5 |*e*| appears in this light not as a fluke of definition but a reflection of a true charge state, and as opposed to the formal oxidation state of Ti. Even in Ti compounds with highly electronegative atoms or moieties, charge self-regulation leads to Ti charge being less than +3, e.g., TiF_4_ (+2.5), TiCl_4_ (+2.7), TiBr_4_ (+2.7), Ti(CN)_4_ (+2.9), Ti(TCNE)_2_ (+2.3) [22]. 

### 3.2. Role of Ligands in the Stabilization of Metal Ions

The stabilization of the charge on a TM center at a value which can be quite far from the FOS implies charge exchange with the ligands. In the examples referenced above, the ligands are bound to the TM by covalent bonds with a degree of ionicity. The stabilization is also at play in coordination complexes formed during solvation of TM cations. In Ref. [23], different transition metal ions in different formal oxidation states solvated by water molecules were studied with DFT and, for selected cases, with wave function-based methods for comparison. The complexes [*M*(H_2_O)_6_]*^n^*^+^ were considered for *M* = V, Mn, Fr, and Cr with *n* ranging from 1 to 5 in the case of V and Fe, 1 to 6 for Cr, and 1 to 7 for Mn (i.e., complexes corresponding to the solvation of V(I) to V(IV), Mn(I) to Mn(VII), Fe(I) to Fe(V), and Cr(I) to Cr(VI) ions. Solvent water molecules participating in the formation of the coordination complex were included explicitly while the rest of the solvent was treated as a polarizable continuum. Transfer of charge density from water molecules coordinated to the TM cation was confirmed, leading to a remarkable charge stability on the TM center regardless of the FOS that were varied in a wide range, similar to what has previously been shown for charges on transition metal cations in inorganic crystals. 

**Figure 6:**
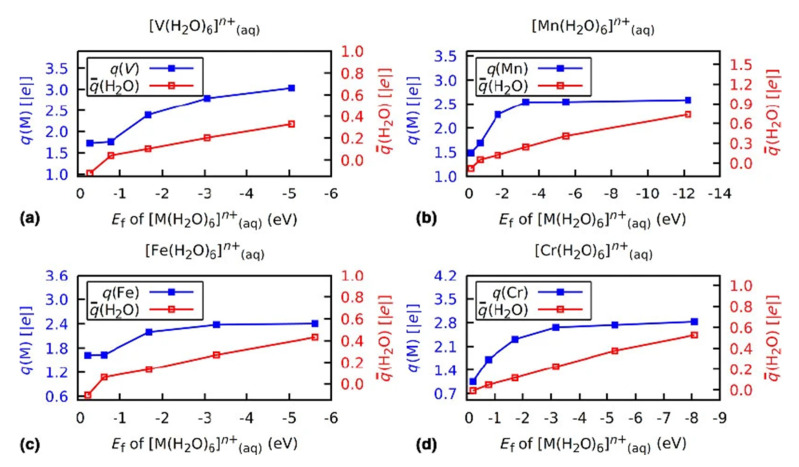
Bader charges on the metal center *q*(M) (blue, filled squares) and average Bader charge on the water ligands $\bar{q}$(H_2_O) (red, empty squares) in [*M*(H_2_O)_6_]*^n^*^+^ complexes with *M* = V (**a**), Mn (**b**), Fe (**c**), and Cr (**d**) versus the formation energy *E_f_* of the corresponding complex. The FOS of the metal center is increasing from left to right (with increasing formation energy) and spans from +I (all cases) to +V (V and Fe), +VI (Cr), and +VII (Mn). These results were obtained with unrestricted DFT calculations using the PBE0 functional and the cc-pVTZ basis set, and a polarizable continuum model. Reprinted with permission from Springer Nature Customer Service Centre GmbH: Springer Nature [23], Copyright 2018.

These results confirm the essence of Pauling’s prediction on the account of solvated TM cations in aqueous solution, made more than 70 years ago and before the appearance of computational chemistry as we know it, that “*the water molecules would accordingly transfer a negative charge of between 2.2 and 3.4 units to the metal atom. This would neutralise the positive charge of the metal atom*” [29]. The correction that computational chemistry makes here is quantitative rather than qualitative, that of relative charge stabilization in an oxidized state, rather than the approximate TM atom neutrality postulated by Pauling. The trends in charges on the TM center and the coordinating water molecules are shown in Figure 6. The range of Bader charges is barely outside the window of $\pm \frac{1}{2}$ predicted by Pauling. 

**Figure 7:**
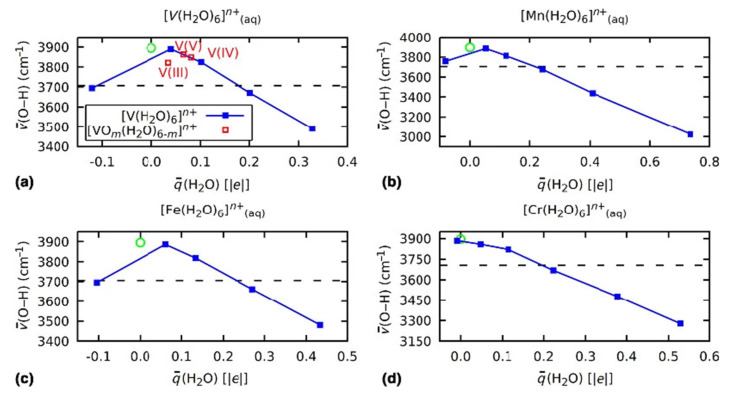
Average O–H stretch frequencies $\bar{\nu }$ of hexaquo transition metal complexes of V (**a**), Mn (**b**), Fe (**c**), and Cr (**d**) with different total charges versus the average H_2_O ligand charge $\bar{q}$ (blue squares). The dashed line indicates the average O–H stretch frequency in a solvated H_3_O^+^, the green circle at $\bar{q}=0$ the average stretch frequency of a solvated H_2_O molecule. The red squares indicate the average ligand charge/frequency pairs for the vanadyl cations [VO(H_2_O)_5_]^+^, [VO(H_2_O)_5_]^2+^, and [VO_2_(H_2_O)_5_]^+^. Reprinted by permission from Springer Nature Customer Service Centre GmbH: Springer Nature [23], Copyright 2018.

This ligand-to-metal charge transfer results in softening of the ligand O–H bonds. Practically, this can be used to explain the formation of higher-FOS transition metalates and oxycations. The average O–H stretching frequency decreases by several hundred cm^−1^ compared to a neutral water molecule at a maximum degree of formal TM oxidation [23]. As can be seen in Figure 7, the average O–H stretch frequencies in the hexaquo complexes show a near-linear dependence on the average ligand charges due to the destabilization of the O–H bond with increasing ionization of the water molecule. This ionization proceeds through the charge donation of the water ligands to the self-regulating TM central ion in the positively charged complexes. Comparison with the average O–H stretch, and therefore bond strength, in H_3_O^+^ suggests that for [*M*(H_2_O)_6_]*^n^*^+^ with high charges *n* > 3, the proton abstraction from water ligands by ambient water molecules in aqueous solution would be favored. This explains the formation of oxovanadyl cations such as [VO(H_2_O)_5_]^+^, [VO(H_2_O)_5_]^2+^, and [VO_2_(H_2_O)_5_]^+^ for vanadium in high oxidation states: the loss of H^+^ reduces the charge loss on the remaining water ligands, which shift back into a more stable ionization/bond strength regime, as indicated in Figure 7. It should be noted that, formally, the water ligands remain at an oxidation state of zero, hence this real-space charge loss cannot be understood in a FOS picture alone. Bader charges on the other hand provide an intuitive way to understand and quantify transition metal complex stabilities with water ligands in aqueous solution. 

### 3.3. Impurity Charge Stabilization

Charge stabilization is also observed in TM dopants or impurities in semiconductors which create bandgap states. Haldane and Anderson analyzed the phenomenon that, contrary to free atoms where different charge states are separated in energy by tens of eV, energy levels of TM impurities in semiconductors which create gap states occur within a rather narrow range within the bandgap for different impurity charge states [21]. This was shown to happen because the actual amount of charge in the core regions of the transition-metal atom is almost the same as in the free atom regardless of the FOS. They showed, using a model Hamiltonian, that the impurity can bind many electrons or holes (i.e., varying considerably the nominal occupancy of d-like states due to hybridization) with the actual charge on the impurity atom itself changing by only a fractional amount, quite in line with Pauling’s prediction. They concluded that whatever the valence, the charge on the impurity remains close to neutrality, with the extra charge localizing in the long hydrogenic tail regions of the impurity wave function.

**Figure 8:**
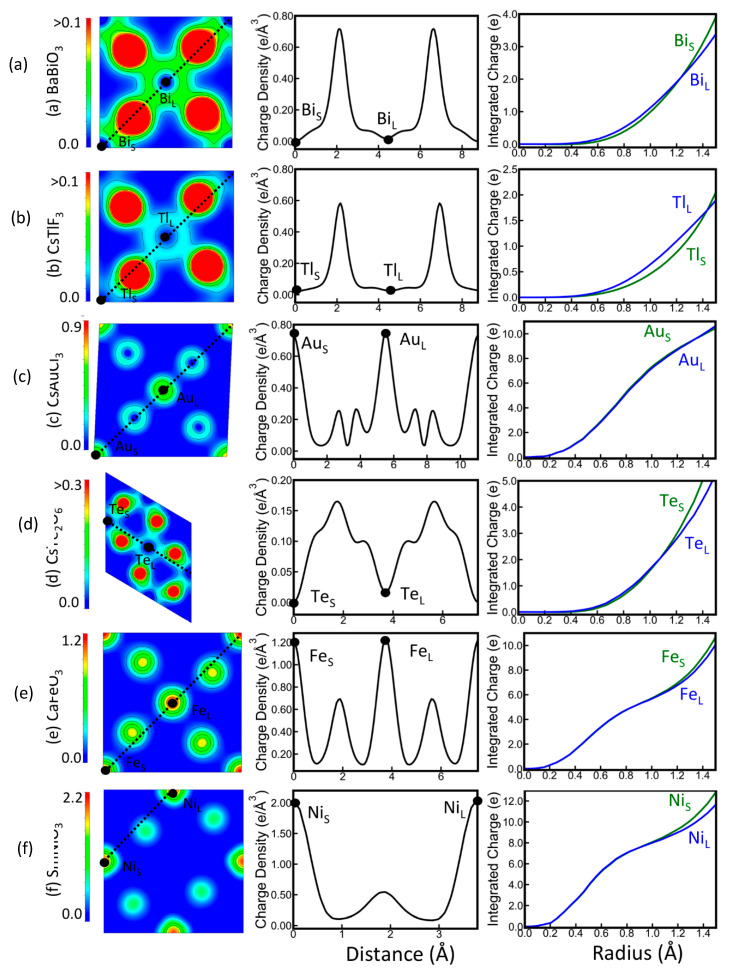
Charge density profiles for the DLE phase of (**a**) BaBiO_3_, (**b**) CsTlF_3_, (**c**) CsAuCl_3_, (**d**) CsTe_2_O_6_, (**e**) CaFeO_3_, and (**f**) SmNiO_3_. The images on the left represent a 2D plot of the total charge density in a plane containing the B atoms in the small (*B*_S_) and large (*B*_L_) octahedra (in units of *e*/Å^3^). The central figures show a 1D plot of the total charge density along the line shown in the figures on the left. The graphs on the right represent the total charge density integrated in a sphere of radius *R* centered on the *B*_S_ and *B*_L_ atoms, as a function of *R* (charge accumulation function). Reprinted with permission from Ref. [20]. Copyright 2018 by the American Physical Society.

Zunger and Lindefelt, in the first ab initio study of this phenomenon considering different types of substitutional TM atoms (from Ti to Zn) in Si, identified a charge self-regulating mechanism ensuring that in such systems the TM can be in many different formal oxidation states corresponding to many different energy levels, all compressed in a narrow energy range [28]. In these earlier and some subsequent works on defects [25], the stability of the charge was considered within a certain radius (of about 2.5 Bohr) rather than within a QTAIM basin. Alippi et al. confirmed charge self-regulation of TM impurities in a DFT study using Bader charge analysis [18]. Dalpian et al. considered *ABX*_3_ perovskites (including BaBiO_3_, CsTlF_3_, CsAuCl_3_, CsTe_2_O_6_, CaFeO_3_, and SmNiO_3,_ the latter two featuring d-electron cations) [20]. They showed that charge self-regulating response is critically important for phenomena such as bond alternation, metal-to-insulator transitions, and the formation of ligand holes, which are typical for this type of compound. These phenomena are related to the possibility of forming in these compounds both different local environments (DLE) and a single local environment (SLE), for the same *B* atom, depending on the phase. The DLE in perovskites is generally associated with a disproportionation reaction, a process commonly rationalized by FOS, where different oxidation states of the same type of atoms in a compound lead to different local geometries. This picture implies a strong connection between FOS and local charge ordering, which in turn drives the local geometry distortions indicative of the disproportionation. However, such a connection could not be found from the charge density analysis in DLE using DFT. Rather than considering Bader charges or charges within a radius, Dalpian et al., similarly to Koch and Manzhos (Section 3.1) [15], directly analyzed the charge density and cumulative charge density up to a radius *R* around the *B* atom. The results are reproduced in Figure 8, where one observes that the physical charge density is almost unchanged around the *B* atom regardless of local environment. In the figures, it is possible to observe that the charge around both *B* atoms in small and large octahedra in DLE compounds (labelled *B*_S_ and *B*_L_) is basically the same despite different formal oxidation states. They conclude that *“the FOS has little or nothing to do with the physical charge density”*. Instead of FOS, the occurrence of DLEs can be explained by the strong hybridization of *X* ligand and *B* metal orbitals forming ligand hole states, a result of the charge self-regulation. Local geometry changes result in stronger or weaker ligand–metal interactions that influence the degree of hybridization between *X* and *B*. Shorter bonds result in higher ligand contributions in small octahedra, increasing the relative energy of these states, while in large octahedra the states around the Fermi level are more metal-dominated and lower in energy. The different metal contributions in the frontier electronic states are again counteracted by changing contributions in lower-lying states, and the total charge around the *B* centers appears constant. This effect results in an energetic stabilization of the disproportionated system and opens a band gap, which was found to be a good predictor for the DLE vs. SLE selection. Similar to the aquo complex stabilities in the preceding section, the use of FOS masks important mechanistic details by not accounting for the details of the charge density distribution in real compounds. This again illustrates how the analysis of charge density distributions between atomic centers can help to unravel the origin of compound stabilities in a coherent way.

### 3.4. Oxygen Redox and PDOS

The redox activity of oxygen in Li-excess transition metal oxide battery materials has been mentioned before and is a recent example of how the FOS concept defines the way redox reactions are rationalized. From a charge-density point of view, oxygen reduction and oxidation, which includes the charge abstraction by formally fully reduced oxide ions, does not come as a surprise given the charge self-regulation of transition metal ions discussed in Section 2.3. However, this needs to be carefully separated from the oxygen redox processes labelled as such based on the FOS analysis, which reflects other material properties than the charge redistribution. This leads to the question if and how the real-space and the FOS electron transfer to oxygen in TMOs are related to each other.

**Figure 9:**
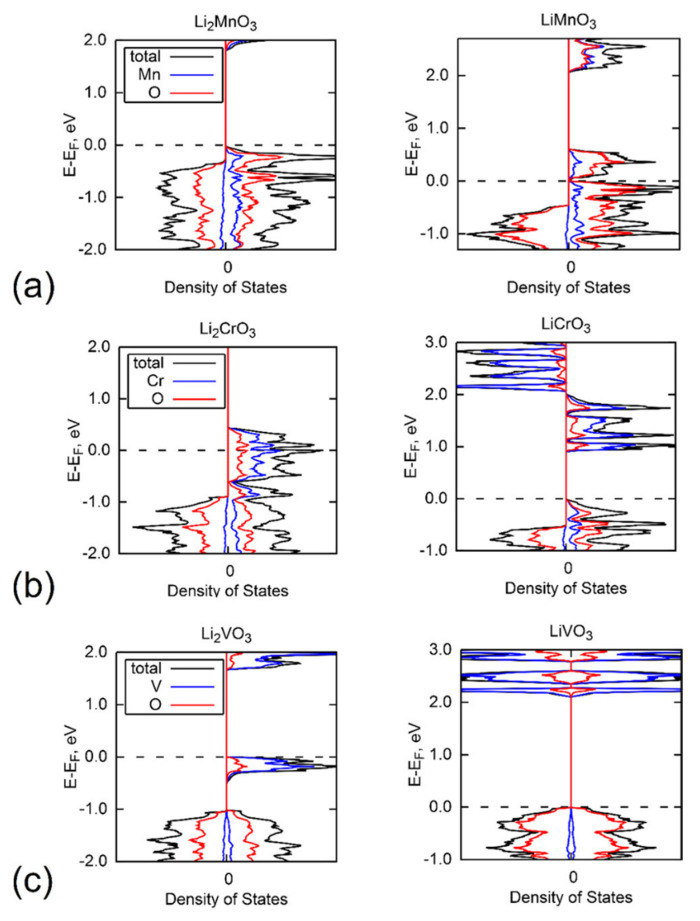
Calculated PDOS of (**a**) Li_2_MnO_3_/LiMnO_3_, (**b**) Li_2_CrO_3_/LiCrO_3_, and (**c**) Li_2_VO_3_/LiVO_3_. The total DOS is shown in black, the vanadium-projected one in blue, and the one for oxygen in red. The Fermi level is indicated by a dashed line and spin-up and spin-down DOS plotted as negative and positive values, respectively. Reprinted with permission from Ref. [24]. Copyright 2020 American Chemical Society.

The recently emerged Li-excess battery electrode materials bear the promise of a higher capacity without a loss in achievable voltages in comparison to currently deployed Li-ion battery cathodes. This is due to additional unoccupied oxygen states in these materials in the charged state, which are introduced by replacement of higher-valent TM ions by Li in the TMO structure, resulting in energetically low-lying O-centered holes. This is reflected in the PDOS of prototypical Li-excess compounds such as LiMnO_3_ which is shown in Figure 9a, where the states at the conduction band minimum are clearly O p dominated and become occupied during lithiation to Li_2_MnO_3_. In contrast, the isostructural LiCrO_3_ and LiVO_3_ show the typical TM d redox upon Li insertion (see Figure 9b,c, respectively) since their TM states lie higher in energy and the formation of TM hole states is preferred over oxygen.

**Figure 10:**
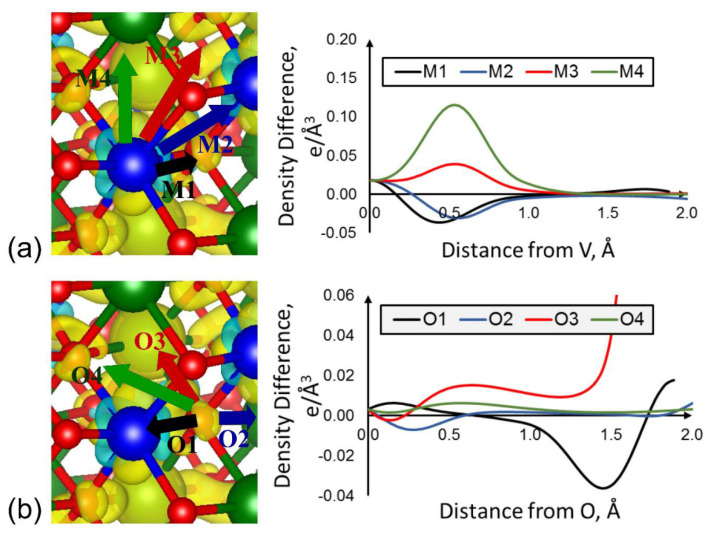
Line profiles (right side) for the charge density difference (left side) between Li2VO3 and LiVO3 around the vanadium (**a**) and oxygen (**b**) centers in different directions. V centers are depicted as blue, oxygen as red, and Li as green spheres. Yellow isosurfaces denote positive, light blue ones negative charge density difference values. Reprinted with permission from Ref. [24]. Copyright 2020 American Chemical Society.

In accordance with the charge self-regulation principle, the Bader charge analysis of these three Li-excess compounds reported in the DFT study in Ref. [24] shows significant changes in the near-oxygen charge density upon reduction for all compounds, while the TM charges are less affected by the electron transfer from Li (which in all cases is almost fully ionized). While quantitatively there is a slightly stronger charge gain on O upon lithiation of the oxygen redox-active LiMnO_3_, the computed charge state changes are overall remarkably similar to LiCrO_3_ and LiVO_3_. From the theory of charge self-regulation, it would be expected that a closer examination of the PDOS of LiCrO_3_ and LiVO_3_, which do not show formal oxygen redox, should reveal a decrease in TM contributions to lower-lying valence states. This would keep the TM-projected DOS integrated over all valence states up to the Fermi level nearly constant in these cases and would result in a charge surplus on O in their reduced form. The integrated PDOS can be expressed as a partial charge and their comparison in Ref. [24] does not show a significant charge gain on O, while there is a more pronounced change for the TM partial charge in LiCrO_3_ and LiVO_3_ upon reduction. It should be noted that for plane-wave basis sets, which are ubiquitously used for DFT computations in the solid state, an appropriate radius for the projection in Equation (3) needs to be set. For computations utilizing the projector-augmented wave (PAW) method [109], the PAW augmentation sphere radii are often used for the PDOS projection, leading to a severe underestimation of the charge present in the cell, especially around O, as shown in Ref. [24], where oxygen partial charges were found positive with the default projection volumes. Increasing the radii to match the Bader basin volumes of the different species improves the total charge within the simulation cell but does not reproduce the same charges and charge state changes found from the Bader analysis. This suggests that there is an inherent blind spot in the way species contributions to electronic states are often determined in the solid state: the charge self-regulation of TMs does not occur in an anisotropic fashion around the TM ions. As previously stated, reduction of TMO compounds leads to a reduction in charge density along the TM–O bond direction, while increasing in the other directions. On the other hand, charge density increase around the large oxide anion with its diffuse orbitals occurs farther away from the nuclei than for the cations. This is schematically shown in Figure 10. As a result, projections around O within a spherical domain are bound to either cut the charge gains short for small radii, or to penetrate into the self-regulation domain of the TM cation, wrongly assigning the charge losses to the oxide ions and cancelling out the charge density increase around them.

**Figure 11:**
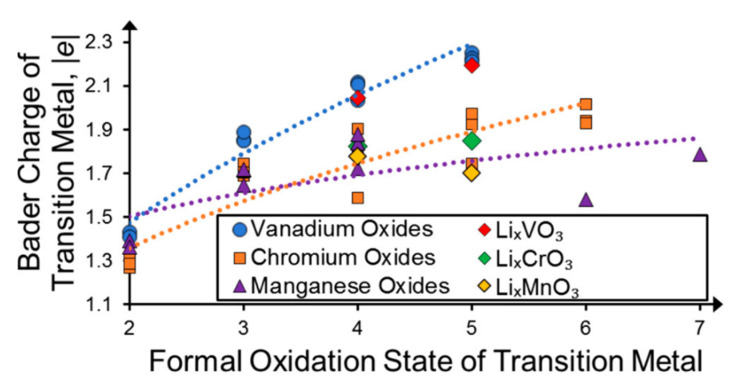
Bader charges of vanadium (blue circles), chromium (orange squares), and manganese (purple triangles) in different binary oxides as well as in their respective Li-excess materials (red, green, and yellow diamonds, respectively) versus the TM FOS. The dotted lines denote least-squares fitted power functions for a clearer distinction of the trends for each TM. Reprinted with permission from Ref. [24]. Copyright 2020 American Chemical Society.

While this shortcoming of the most common way the PDOS is obtained in literature does not change the FOS picture, it does bias the electronic state representation towards TM redox and hides the contribution shifts of TM ions during oxidation and reduction from a closer analysis, which might have aggravated the sparse acknowledgment of the TM self-regulation effect in literature. In the grand picture, it can be stated that oxygen redox activity is, from a *charge density point of view*, widespread in transition metal oxides beyond Li-excess or other compounds with unoccupied O-centered states at the Fermi level. Similar to Figure 6 for TM aquo complexes, the nearly constant TM Bader charges over a wide range of oxidation states can also be found in crystalline TMOs, as shown in Figure 11 for vanadium, chromium, and manganese oxides, over a wide range of TM/O ratios in accordance with the charge self-regulation picture. The TM Bader charges of the Li-excess compounds agree well with the overall trends of their respective binary oxides. The TMO Bader charges are also of a similar magnitude to the Bader charges found for other TMOs, TM aquo complexes, and molecular TM halides discussed in the previous sections, generally ranging between +1 and +3. While the FOS predicts TM cations in most instances as the redox-active species, it becomes apparent that their ligands mediate any charge density changes to a significant degree, irrespective of whether their participation in the redox process is formally expected or not. 

## 4. Conclusions

The concept of formal oxidation states has heavily influenced the conceptual understanding of matter and of phenomena. As a construct based on simple combinations of simple approximations to electronic states, it is bound to have blind spots and limits to its applicability. It often allows for reliable predictions with regard to structures, and it always, albeit to different degrees depending on the application, misrepresents the mechanism of redox reactions involving transition metal atoms. That mechanism is subject to the principle of charge self-regulation, which leads to relatively small changes of physical electron density around the TM atom regardless of the oxidation state. This principle was, in a very general form, already expressed by Linus Pauling in 1948, confirmed in various works in the 1970s and 1980s, and firmly established only in this century. The language of formal oxidation states has, over the years, considerably colored the perception of the mechanism of redox reactions. One consequence of this is the underappreciation of the extent and ubiquitous nature of anionic redox activity, a mechanism which attracts particular attention in metal ion batteries. One can still think in terms of electrons being donated to and reducing the TM center. According to the principle of charge self-regulation, this would correspond to less charge drawn from the ligands (which was clearly illustrated in solvated complexes described in Section 3.2). From a charge density perspective, this is still effectively oxygen redox, as significant charge redistribution occurs around the ligands.

This perspective is supposed to serve as a reminder of the caveats when rationalizing redox processes involving transition metal compounds, which are a prominent subject of research in numerous fields in science and engineering. While the FOS concept is rarely employed for covalent compounds, e.g., for the understanding of organic reactions, since its predictive and descriptive power is limited for such compounds, and while it works very well if the bonding is predominantly ionic, many transition metal compounds are found between those extremes. Therefore, density-based descriptors of charge states and charge transfer can help to augment the FOS formalism for a more complete and more realistic picture. The covalent character of TMOs and the significant charge remainder on formally fully oxidized TM cations due to their charge self-regulation was exemplified by TiO_2_ in Section 3.1. In Section 3.2 and Section 3.3, the advantages of a density-based discussion of redox reaction mechanisms and TM compound properties were demonstrated on aqueous TM complexes and in the solid state for TM impurities or perovskites, respectively. Lastly, Section 3.4 elucidated possible reasons why charge self-regulation might not be well-recognizable when analyzing electronic states in extended systems and discussed the meaning of anionic redox in electrochemical energy storage in the FOS and charge density picture.
